# COVID-19 Vaccination Acceptance among Healthcare Staff in Sudan, 2021

**DOI:** 10.1155/2022/3392667

**Published:** 2022-02-09

**Authors:** Eman Omer Mohamed Yassin, Haybat Awad Allah Faroug, Zainab Bushra Yousif Ishaq, Mustafa Mohammed Alfaki Mustafa, Mohammed Meshal Abdulkareem Idris, Samah Elnour Khalifa Widatallah, Ghada Omer Hamad Abd El-Raheem, Maha Y. Suliman

**Affiliations:** National University, 11114, 11111 Khartoum, 79371, Sudan

## Abstract

Elderly and patients with comorbid conditions have higher risk of infection and complications. Vaccination hesitancy is defined as the refusal of vaccine or the delay in accepting it despite the availability of vaccines and vaccination services. This study was aimed at assessing knowledge, perception, and acceptability of healthcare staff towards different types of COVID-19 vaccination. A multicenter hospital-based descriptive cross-sectional study was implemented to study the knowledge, perception, and acceptability of healthcare staff towards COVID-19 vaccination. Multistage sampling technique was applied. Data were collected through a self-administered questionnaire filled by the participants. 400 participants were studied. 61% of the participants were females, and the most frequent age reported was between 18 and 35 years (67%). A statistically significant association (*p* = 0.048) was found between knowledge about vaccination and professions. The most common vaccine type known and accepted was AstraZeneca vaccine. On assessing acceptability of COVID-19 vaccination, acceptance rate was high (63.8%) and 22.7% of the participants had already got vaccinated. The rejection rate among our staff was 27.4%. This study was conducted in April, 2021. Majority of our healthcare staff believed that vaccination is the key to combat the pandemic. One of the issues and concerns about vaccination was the safety and the risk of developing acute adverse events (*p* = 0.001). Encouraging factor for vaccination was the fear of getting infection themselves and their families. The present study revealed the presence of good knowledge and acceptability among medical staff towards COVID-19 vaccinations in Sudan.

## 1. Introduction

COVID-19 infection can cause fatal pneumonia. Elderly and patients with comorbid conditions have a higher risk of infection and complications, such as acute respiratory distress syndrome (ARDS) and cytokine storm [1–3].

There are many types of vaccines. Whole virus vaccines contain viruses whose genetic material has been destroyed by heat, chemicals, or radiation. These whole virus vaccines can be either live attenuated or inactivated. Another type of vaccine is the subunit vaccine, containing purified pieces of the virus selected specially for their ability to stimulate immune cells. Only protein subunit vaccines are being developed against the virus that causes COVID-19. Nucleic acid vaccines use the genetic materials of the pathogen to stimulate the immune response [3]. In the case of COVID-19, spike proteins of the surface of the virus interact with human cells to trigger an immune response. After the infection, the body keeps a few T-lymphocytes, called memory cells, that go into quick action upon reinfection [4–6]. Current types of COVID-19 vaccines are mRNA vaccines, protein subunit vaccines, and vector vaccines [4–6].

Vaccination is considered as one of the most effective methods for controlling infectious diseases [7]. Moreover, the vaccination programs contributed remarkably in the reduction of morbidity and mortality of many infectious diseases. Despite this, the success of vaccination faces challenges from groups and individuals who delay or refuse vaccination [8, 9]. Vaccination hesitancy is defined as the refusal of vaccine or the delay in accepting it despite the availability of vaccines and vaccination services [10]. Thus, encouragement by policy makers should be implemented to increase the vaccination willingness [11].

In Sudan, as of Jan. 2022, 13^th^, the number of suspected cases was 93,390 and 50,621 confirmed cases of COVID-19 reported to the Federal Ministry of Health [12]. COVID-19 detection at community level program was developed by the FMOH. This program was called “Syndromic Approach.” In this program, community volunteers detect and report COVID-19 cases, each volunteer was responsible for 150 households, and the volunteer had 50 visits per day and conducted second rounds every 3 days. Each 40-50 volunteers were supervised by a rapid response team that decides whether the infected populations need home isolation or hospital isolation. On the other hand, the vaccination status as per the reports of the Federal Ministry of Health (FMOH) was published in the official website (http://sho.gov.sd/corona/). Khartoum state was the first state to get the vaccination services. The vaccination program firstly targeted the elderly (>60 years) and the healthcare workers. Between the period of 28^th^ of March and 5^th^ of April, 34806 elderly populations (>60 years) were vaccinated and among healthcare workers, 23266 were vaccinated. This study was aimed at assessing knowledge, perception, and acceptability of healthcare staff towards different types of COVID-19 vaccination.

## 2. Methods

A multicenter hospital-based descriptive cross-sectional study was implemented to study the knowledge, perception, and acceptability of healthcare staff towards COVID-19 vaccination. The study assessed the characteristics of healthcare staff who worked at governmental hospitals in Khartoum state, as well as their acceptability to take COVID-19 vaccination. Based on the reports of the Federal Ministry of Health in Sudan (http://sho.gov.sd/corona/), vaccination centers were installed in Khartoum state targeting, at first level, the healthcare workers and elder population. Khartoum state is the capital of Sudan located at LAT 15.46414700 and LON 32.47161300. It is the most populated state in Sudan and has more advanced healthcare services than other states.

A multistage sampling technique was applied to select the participants. At first level, 8 tertiary governmental hospitals in Khartoum state were randomly included in the study. All were multispecialty hospitals located in Khartoum state. Ibn Sina Hospital, Ibrahim Malik Hospital, Khartoum Hospital, Soba University Hospital, Haj Elmardi Mohieldin Teaching Hospital, Bashair University Hospital, Alturkey Hospital, and Alribat University Hospital were included in the study. At second level, stratified random sampling technique was used to select healthcare staff working in the governmental hospitals based on the size of each hospital; the selection from the different hospitals corresponded to the percentage contributed by the hospital to the population using the formula of sample size estimation for each stratum (hospital) was *n* = *N*/1 + *Nd*^2^, where *n* is the estimated sample size, *N* is the total number of patients records, and *d* is the degree of accuracy set at 0.05. The total sample size collected from all 8 hospitals with respect to the size of each hospital was 400 healthcare workers of different professions. Data were collected through a self-administered questionnaire filled by the participants on-site after getting their approval through informed consents. This tool was developed by two co-authors and validated by a research specialist prior to data collection. The response rate to this self-administered questionnaire was 100% with no dropouts, yet some of the questions were not answered by all participants (detailed in the tables). All the questions were closed questions, either straight forward questions (for the demographic characteristics) or check list (for knowledge and perception). The Statistical Package for Social Sciences (SPSS version 23) was used to describe and analyze the data. Chi-square test was done to assess the differences between variables; it was considered statistically significant when *p* ≤ 0.05. Confidentiality of participants was assured through the use of an anonymous research tool with no identifiers. Ethical approval from the Ministry of Health was granted firstly; then, approval from each hospital administration was granted. The collected data were used strictly for the purpose of the study objectives. This study was conducted in April, 2021. The data were collected in a 2-month period covering all the study area and population. This study was done in Khartoum state because at the time of the study, Khartoum state was the first state that started the vaccination services. Other states had started after this state.

## 3. Results

### 3.1. Characteristics of the Study Participants

Four hundred participants from different governmental hospitals were assessed in our study; all healthcare workers in the facility were included. 61% of the participants were females, and the most frequent age reported was between 18 and 35 years (67%); only 2% were aged above 55 years. Regarding their professions, physicians and nurses were the majority (31% and 28%, respectively), 15% were pharmacists, while other professions are mentioned in Table 1. Chronic conditions of the participants are described in Table 1. 84.5% had no chronic condition. With regard to their general vaccination status, 30% of our participants had received vaccination of any type during the year of 2020.

### 3.2. Assessment of Knowledge of Participants about COVID-19 Vaccination Based on Their Professions

Based on the different professions of the participants, their knowledge was assessed based on their professions. 365 participants have reported their knowledge about COVID-19 vaccines. From the participants who were aware of COVID-19 vaccination, 47.7% were nurses and technicians, while doctors were 35.7%, and pharmacists were only 16.5%. A statistically significant association (*p* = 0.048) was found between knowledge about vaccination and professions.

Regarding knowledge of participants about vaccination safety and number of vaccine shots, 359 and 367 participants have replied. No statistically significant difference in knowledge was found among the different professions (*p* = 0.404 and 0.152, respectively).  Table 2 illustrates the details.

### 3.3. Sources of Information about COVID-19 Vaccination as Reported by Participants

**Figure 1:**
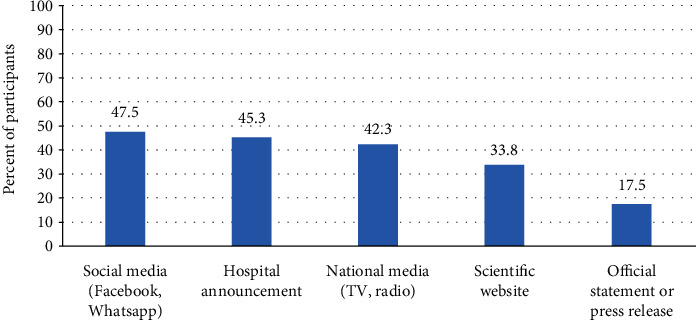
Sources of information about COVID-19 vaccines as reported by participants (*n* = 400).

Participants were asked to report the sources of their knowledge about COVID-19 vaccination. The most frequently reported sources of information were the social media and the hospital announcement, reported by 47.5% and 45.3% of the participants, respectively, followed by the national media as TV and radio (42.3%). 33.8% of the participants had their knowledge from scientific websites, while 17.5% of the participants had their knowledge from official statements and press release (Figure 1).

### 3.4. Knowledge of Participants about the Types of COVID-19 Vaccines

**Figure 2:**
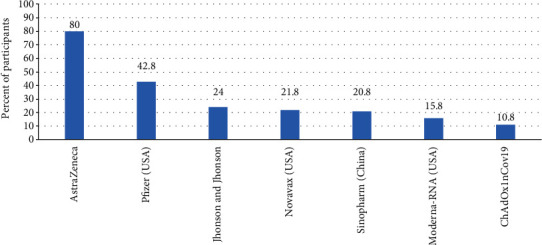
Knowledge of participants about types of COVID-19 vaccines (*n* = 400).

Different types of COVID-19 vaccination were reported by the participants, through a closed checklist question answered by each participant, selecting each known vaccine to them. The most common vaccine type known by the participants was AstraZeneca, known by 80% of the participants, followed by Pfizer vaccine (43%). Other types of vaccines and the percent of participants who knew them are detailed in Figure 2.

### 3.5. Assessment of the Perception of Participants about COVID-19 Vaccination Based on Their Professions

From the participants who believe that vaccination is the key to stop the pandemic, 50.6% were nurses and technicians, while 32.8% were doctors, and 16.6% were pharmacists. No statistically significant difference was found between the perception of participants about vaccination as a key to stop the pandemic and different professions of participants (*p* = 0.716).

Regarding the opinion of participants about vaccination for either COVID-19 recovered or infected people, no statistically significant difference was found between the perception of participants and their professions (*p* > 0.05) (Table 3).

### 3.6. Perception of Participants about Criteria of Population That Should Get COVID-19 Vaccination

**Figure 3:**
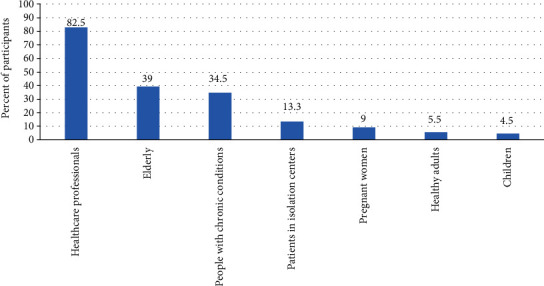
Perception of participants about criteria for vaccination (*n* = 400).

83% of our study population reported that healthcare workers were the group of population that should get vaccinated, followed by elderly population and people with chronic conditions (39% and 35%, respectively). Other categories are detailed in Figure 3.

### 3.7. Acceptability of the Participants regarding COVID-19 Vaccination Based on Their Professions

Acceptability of COVID-19 vaccination was assessed among different professions of our study population. Of the participants who accepted COVID-19 vaccination, 45.1% were nurses and technicians, while 38.2% were doctors, and 16.7% were pharmacists. A statistically significant association was found (*p* = 0.032) between vaccine acceptability and professions. Regarding encouraging family members, no statistically significant association was found between encouraging family and professions (*p* > 0.05).

The time which the participants were intending to get vaccinated was assessed among different professions of the participants. Of those who already took COVID-19 vaccine, 50% were doctors, 34.1% were nurses and technicians, while 15.9% were pharmacists. Among the participants who intended to get COVID-19 vaccination as soon as possible, 50% were nurses, 33.9% were doctors, and 16.1% were pharmacists. Of those who are not intending to get vaccinated at all, 65.7% were nurses, 20.2% were doctors, and 14.1% were pharmacists. A statistically significant association (*p* = 0.001) was found between intention of getting vaccination and professions of participants (Table 4).

### 3.8. Association between Acceptability of COVID-19 Vaccination and the Characteristics of the Participants

Based on characteristics of participants, acceptability of COVID-19 vaccination was assessed. No statistically significant association was found between the characteristics of participants, as gender, age, chronic conditions, any vaccination before, and their acceptability of COVID-19 vaccination (*p* > 0.05). However, there was a statistically significant association between the safety of vaccination and the acceptance of participants to get it (*p* = 0.001).  Table 5 illustrates the associations between characteristic of the participants and their acceptability of COVID-19 vaccination.

### 3.9. Types of COVID-19 Vaccines Accepted to Be Taken by the Participants

**Figure 4:**
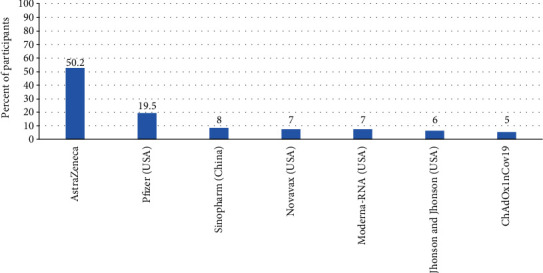
Types of vaccines accepted to be taken by the participants.

Different types of vaccines that were accepted to be taken by the participants are reported in Figure 4. The most accepted type of vaccine was AstraZeneca, accepted by 50% of the participants, followed by Pfizer vaccine, accepted by 20% of the participants. Sinopharm (China) vaccine was accepted by 8%. Other types of vaccines accepted by the participants are mentioned in Figure 4.

### 3.10. Factors Affecting the Choice of COVID-19 Vaccines as Reported by the Participants

**Figure 5:**
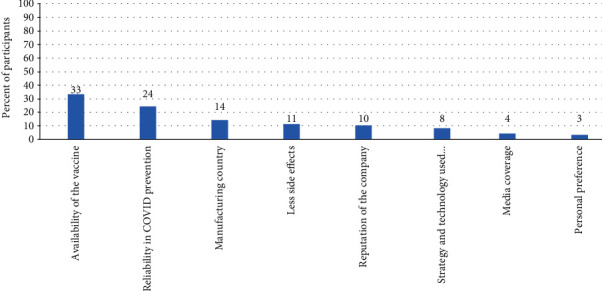
Factors affecting the choice of COVID-19 vaccines as reported by the participants.

These types of vaccines chosen above were preferred by the participants based on various factors. The most frequently reported factor affecting the choice of vaccine type was the availability of the vaccine in Sudan (reported by 33% of the participants), followed by the reliability of the vaccine in preventing COVID-19 infection (24%). The manufacturing country was the third frequently reported factor (14%). Less side effects were the factor affected the choice of 11% of the participants. Other factors are mentioned in Figure 5.

### 3.11. Reasons for Either Taking COVID-19 Vaccine or Not as Reported by the Participants

The reasons reported by the participants for weather taking the vaccine or not were detailed below, and their frequency was detailed.

### 3.12. Reasons behind Accepting Taking COVID-19 Vaccines Available in Sudan

**Figure 6:**
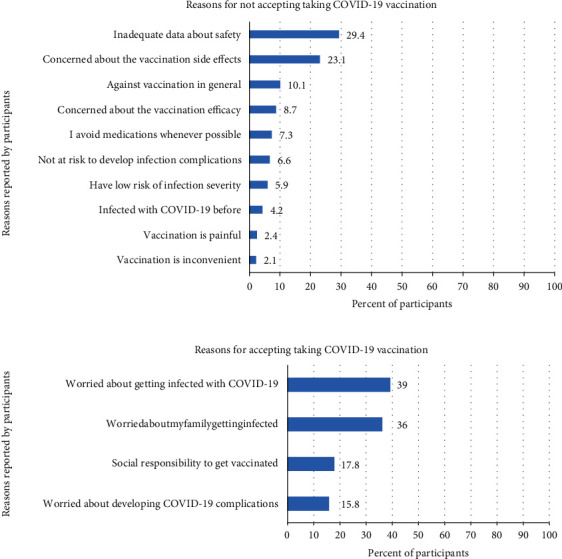
Reasons behind not taking COVID-19 vaccines as reported by participants (*n* = 400).

The most common reasons reported by the participants behind accepting to get COVID-19 vaccination were the worry about getting infected or a family member getting infected. These two reasons were reported by 39% and 36% of the participants, respectively. Other reasons for accepting vaccination were the social responsibility (17.8%) and worry about COVID-19 complications (15.8%). On the other hand, 4% of the participants had no intention to get COVID-19 vaccine (Figure 6).

### 3.13. Reasons for Not Taking COVID-19 Vaccines as Reported by the Participants

On the other hand, the reasons for rejecting COVID-19 vaccines were recorded. The most frequently reported reasons were the inadequate data about safety and the concern about side effects. These reasons were reported by 29.4% and 23.1% of the participants who rejected the vaccination. Other reasons are detailed in Figure 6.

## 4. Discussion

Most of the healthcare staff in this study were young (67%) and aged between 18 and 35 years. This percent was slightly higher than that of the medical staff in United Arab Emirates (50.8%) and lower than the percentage of young staff in Saudi Arabia (72.1%) [13, 14], while in other studies, less than 30% of their staff were aged below 30 years [14–17]. No statistically significant difference was found (*p* = 0.13) between the age of our participants and their acceptability of COVID-19 vaccination, contrary to the Turkish and Chinese community members (*p* = 0.001) with older ages being more willing to get COVID-19 vaccines [18, 19].

Females comprised 61% of the healthcare workers in our study, similar studies [20–22] and contrary to the Emirati staff who had more males (64.9%) than females [13]. However, in other studies [14–17, 23, 24], percentages of males and females were almost equal. There was no statistically significant difference in vaccine acceptability between males and females in our study (*p* = 0.544) as in the study of Saied et al. in Egypt (*p* = 0.263) [21]. However, in a Turkish study, males were more willing to get COVID-19 vaccination than females (*p* = 0.02) [18].

Having chronic conditions had no statistically significant association (*p* = 0.638) to vaccine acceptability as in Turkey (*p* = 0.397) [18].

Lab technicians and pharmacists were the minority of the medical staff (17.8% and 15%, respectively) compared to doctors and nurses (31% and 28%, respectively).

In Sudan, the medical staff had higher awareness about COVID-19 vaccines (91.2% of the staff) compared to published literature (65.7% and 65.8%) [14, 22], with a statistically significant difference (*p* = 0.048) among different professions, with doctors being the most aware population.

Sources of information play an important role in vaccination knowledge and acceptability [25]. Our staff reported that the primary source of information about COVID-19 vaccination was the social media as Facebook and WhatsApp (47.5%). This was a preferred source among Egyptian health students [21]. However, in another study, the primary source of information was the government website (46%) and the social media was the source for only (17%) of their staff [13].

Regarding perception of COVID-19 vaccination, 72.8% of our healthcare staff believed that vaccination is the key to combat the pandemic, and lower percentage (67.9%) was reported in an Egyptian study [21]. Even though 5.9% of our staff believed that they were at low risk of getting severe infection, this belief was common (22.4%) among Egyptian staff [19]. By far, the most known vaccine among our staff was AstraZeneca vaccine (80%), followed by Pfizer (43%); other vaccines were less known.

Positively, 83% of our study population reported that healthcare workers were one or the priority groups of population that should get vaccinated, followed by elderly population and people with chronic conditions (39% and 35%, respectively). This was in line with the WHO, 2020 Strategic Advisory Group of Experts [26].

On assessing acceptability of COVID-19 vaccination, acceptance rate was high (63.8%), similar to the rate in China [19] and higher than reported rates (19.6%-60%) in other studies [20, 23, 27–30]. With regard to profession, nurses and lab technicians were the most accepting group (45.1%), followed by doctors (38.2%). The least accepting group was the pharmacists (16.7%) (*p* = 0.032). Furthermore, in a Canadian study, acceptance of COVID-19 vaccination was the highest among physicians (95%), followed by nurses and administrative staff [20].

The most prevalent reason encouraging vaccination among our staff was the worry of getting COVID-19 infection themselves (39%) or their families (36%). These percentages were lower than reported (47%) in UAE [13]. To put in mind, the percentages depend on when the studies were done, as most countries have ongoing vaccination programs. On the other hand, worry about families was the most prevalent reason among Lebanese and Egyptian health staff (80% and 77.7%, respectively) followed by worrying about themselves (78% and 35.1%), respectively [15, 21].

At the time of the study, 22.7% of the participants had already got vaccinated, with doctors being the most vaccinated group (50%), followed by nurses and technician (34.1%). In China, 34.4% of the participants had received COVID-19 vaccination [19]. This percentage of vaccinated staff was higher than the reported percent (15%) in UAE [13]. On the other hand, pharmacists who got vaccinated were (15.9%) (*p* = 0.001). Additionally, the most accepted type of vaccination among our healthcare staff was AstraZeneca vaccine, accepted by 50% of the participants, followed by Pfizer vaccine (20%). In contrast to that, in Egypt, the most accepted vaccine was Pfizer (22%) followed by AstraZeneca (7.1%) [21]. Moreover, the most accepted vaccines in Brazil were USA vaccine (82%) and Oxford/England vaccine (81%). Nevertheless, China vaccine was favored by only 8% of our participant, while in Brazil, it was accepted by 67% of their participants [31]. Availability of the vaccine was the most prevalent factor affecting the choice of COVID-19 vaccines as reported by the participants (33%) followed by reliability in protection (24%) and lesser side effects (11%). The same as in Egypt, the motivations for getting COVID-19 vaccines were the effectiveness (14.2%) and the availability (11.7%) [21]. The acceptance of a particular brand of COVID-19 vaccination would depend on the availability and on the national policy of administration of vaccines as well. By the time of the study, only AstraZeneca vaccine was available in vaccination centers, but other vaccines were arriving.

One of the issues and concerns about vaccination was the safety and the risk of developing acute adverse events [19, 30, 32–34]. Fear of adverse effects was expressed by 23.1% of our participants and by 56.3% of Egyptian participants reported in a published study [21]. Vaccination safety was an issue among 53.5% of our participants; this percent was lower in some studies [17, 21, 35]. Nevertheless, these barriers were the reported barriers among Lebanese health staff with the concern about side effects being the most reported barrier (64%) [15]. Moreover, in a multinational study, acceptance of vaccines with minor adverse reactions was the reason reported with a percentage ranging from 42% to 86.4% based in the country [16]. Other reported reasons among our staff were as follows: have low risk for developing severe infection (5.9%), have natural immunity from previous infection (4.2%), and dislike of injections (2.4%). These same reasons were reported in a Canadian study with comparable percentages [20].

The rejection rate among our staff was 27.4%, similar to published studies [17, 21]. Contrary to that, the rejection rate was mush lower in other studies [19, 20, 23, 35]. Among those rejecting vaccination, 65.7% were nurses and technicians, 20.2% were doctors, and 14.1% were pharmacists (*p* = 0.001). Vaccination safety had a statistically significant association to the acceptability of vaccines among our staff (*p* = 0.001). This strong association (*p* < 0.001) was reported by İkiışık et al. and Saied et al. as well [18, 21].

Since majority of health staff work at public hospitals [22], our study assessed the staff in governmental hospitals to get a clear image about vaccination hesitancy. The privilege of our study is that it tangled the vaccination acceptance among different professions of healthcare staff (doctors, pharmacists, lab technicians, nurses, and administrative staff).

Our study was not without limitations; the data collected from working staff were not validated through Cronbach test of reliability. Moreover, private sector was not covered in this study. Moreover, wider studies covering all states of Sudan, as well as community-based studies, will give a clearer picture about vaccination knowledge and acceptability.

## 5. Conclusion

The present study revealed the presence of good knowledge and acceptability among medical staff towards COVID-19 vaccinations in Sudan. The healthcare staff in Sudan were mostly young in their age more than elsewhere. As in many other countries, concern about safety and side effects governed the vaccination acceptance. Majority of our study population had a perception that healthcare workers were the group of population that should get vaccinated first, followed by elderly population and people with chronic conditions. The mostly known and accepted COVID-19 vaccines were AstraZeneca and Pfizer. This might be dependent on the availability and on the national policy of administration of vaccines. Encouraging factor for vaccination was the fear of getting infection themselves and their families.

## 6. Recommendations

The construction of immediate health educational programs and more accurate information should be distributed and advertised by respective health authorities for all community members. Policy makers should take steps to ensure encouragement, positive perceptions, and improved acceptability towards COVID-19 vaccinations in order to reduce the vaccine hesitancy. In Sudan, using the social media to spread the correct knowledge is an important approach as it was the primary source used by healthcare workers as shown by this study. More vaccination centers or community vaccination teams will fasten the vaccination process and provide more coverage. Moreover, more studies similar to this study are needed in other states of Sudan.

## Figures and Tables

**Table 1:** Characteristics of the study participants (*n* = 400).

Characteristics	*n*	%	Characteristics	*n*	%
Gender			Chronic conditions		
Male	156	39	None	338	84.5
Female	244	61	Cardiovascular conditions (HTN, heart disease)	14	3.5
Age			Diabetes mellitus	18	4.5
18-35 years	268	67	Asthma, COPD	10	2.5
36-55 years	124	31	Others	20	5
>55 years	8	2	Any vaccination taken last year		
Professions			Yes	120	30
Physician	124	31	No	280	70
Pharmacist	60	15			
Nurse	112	28			
Lab technician	71	17.8			
Administrator	19	4.8			
Others	14	3.5			

**Table 2:** Assessment of knowledge of participants about COVID-19 vaccination based on their professions.

	Professions								
Knowledge	Doctors	%	Pharmacists	%	Nurses/technicians	%	Total	%	*p* value
Are you aware of COVID-19 vaccines									
Yes	119	35.7	55	16.5	159	47.7	333	91.2	0.048^∗^
No	5	15.6	5	15.6	22	68.8	32	8.8	
Total	124	34.0	60	16.4	181	49.6	365	100.0	
COVID-19 vaccines safety									
Not safe	71	37.0	28	14.6	93	48.4	192	53.5	0.404
Safe	52	31.1	31	18.6	84	50.3	167	46.5	
Total	123	34.3	59	16.4	177	49.3	359	100.0	
Number of vaccines shots									
One shot	12	31.6	6	15.8	20	52.6	38	10.4	0.152
Two shots	104	35.3	52	17.6	139	47.1	295	80.4	
More than two shots	7	25.9	2	7.4	18	66.7	27	7.4	
Do not know	1	14.3	0	0.0	6	85.7	7	1.9	
Total	124	33.8	93	25.3	183	49.9	367	100.0	

^∗^Statistically significant.

**Table 3:** Assessment of perception of participants about COVID-19 vaccination based on their professions.

	Professions								
Perception	Doctors	%	Pharmacists	%	Nurses/technicians	%	Total	%	*p* value
Is vaccination the key to stop the pandemic									
Yes	87	32.8	44	16.6	134	50.6	265	72.8	0.716
No	37	37.4	15	15.2	47	47.5	99	27.2	
Total	124	34.1	59	16.2	181	49.7	364	100.0	
Vaccination for recovered people from COVID-19									
Yes	81	36.0	36	16.0	108	48.0	225	61.3	0.801
No	23	28.8	13	16.3	44	55.0	80	21.8	
Do not know	20	32.3	11	17.7	31	50.0	62	16.9	
Total	124	33.8	60	16.3	183	49.9	367	100.0	
Vaccination for COVID-19 infected people									
Yes	46	29.9	30	19.5	78	50.6	154	42.0	0.389
No	57	39.0	19	13.0	70	47.9	146	39.8	
Do not know	21	31.3	11	16.4	35	52.2	67	18.3	
Total	124	33.8	60	16.3	183	49.9	367	100.0	

**Table 4:** Acceptability of the participants regarding COVID-19 vaccination based on their professions.

	Professions								
Acceptability	Doctors	%	Pharmacists	%	Nurses/technicians	%	Total	%	*p* value
Accepting to get vaccinated									
Yes	89	38.2	39	16.7	105	45.1	233	63.8	0.032^∗^
No	34	25.8	21	15.9	77	58.3	132	36.2	
Total	123	33.7	60	16.4	182	49.9	365	100.0	
Encourage family to take COVID-19 vaccine									
Yes	94	38.2	39	15.9	113	45.9	246	68.1	0.135
No	30	26.8	17	15.2	65	58.0	112	31.0	
Do not know	0	0.0	1	33.3	2	66.7	3	0.8	
Total	124	34.3	57	15.8	180	49.9	361	100.0	
When are you going to get vaccinated									
Already did	41	50.0	13	15.9	28	34.1	82	22.7	0.001^∗^
As soon as possible	42	33.9	20	16.1	62	50.0	124	34.3	
After few months	20	35.7	12	21.4	24	42.9	56	15.5	
Never	20	20.2	14	14.1	65	65.7	99	27.4	
Total	123	34.1	59	16.3	179	49.6	361	100.0	

^∗^Statistically significant.

**Table 5:** Association between acceptability of COVID-19 vaccination and the characteristics of the participants.

	Accepting to get vaccinated						
Characteristics	Yes	%	No	%	Total	%	*p* value
Gender							
Male	102	65.8	53	34.2	155	39.0	0.544
Female	152	62.8	90	37.2	242	61.0	
Total	254	64.0	143	36.0	397	100.0	
Age							
18-35 years	163	61.0	104	39.0	267	67.3	0.13
36-55 years	85	69.1	38	30.9	123	31.0	
>55 years	6	85.7	1	14.3	7	1.8	
Total	254	64.0	143	36.0	397	100.0	
Professions							
Physician	89	72.4	34	27.6	123	31.0	
Pharmacist	39	65.0	21	35.0	60	15.1	0.127
Nurse	67	60.4	44	39.6	111	28.0	
Lab technician	38	53.5	33	46.5	71	17.9	
Administrator	13	72.2	5	27.8	18	4.5	
Others	8	57.1	6	42.9	14	3.5	
Total	254	64.0	143	36.0	397	100.0	
Chronic conditions							
Yes	40	66.67	20	33.33	60	15.11	0.638
No	214	63.5	123	36.5	337	84.89	
Total	254	63.98	143	36.02	397	100	
Received any vaccination last year							
Yes	79	69.3	35	30.7	114	29.1	0.207
No	174	62.6	104	37.4	278	70.9	
Total	253	64.5	139	35.5	392	100.0	
COVID-19 vaccine safety							0.001^∗^
Not safe	120	57.4	89	42.6	209	54.0	
Safe	131	73.6	47	26.4	178	46.0	
Total	251	64.9	136	35.1	387	100.0	

^∗^Statistically significant.

## Data Availability

All supporting data are available.

## Abbreviations

ARDS: Acute respiratory distress syndrome
FMOH: Federal Ministry of Health
MOH: Ministry of Health
mRNA: Messenger ribonucleic acid
WHO: World Health Organization.

## Supplementary Materials

Supplementary Materials Table S1: knowledge of participants about COVID-19 vaccination. Table S2: perception of participants about COVID-19 vaccination. Table S3: acceptability of participants for COVID-19 vaccination. Table S4: reasons behind accepting taking COVID-19 vaccines available in Sudan. Table S5: reasons behind not taking COVID-19 vaccines as reported by participants.
