# Supplementary material for: COVID-19 Vaccination Acceptance among Healthcare Staff in Sudan, 2021
Source: J Immunol Res. 2022 Feb 9;2022:3392667. doi: 10.1155/2022/3392667 (PMC8832156; doi:10.1155/2022/3392667)
Supplement: Supplementary Materials — Table S1: knowledge of participants about COVID-19 vaccination. Table S2: perception of participants about COVID-19 vaccination. Table S3: acceptability of participants for COVID-19 vaccination. Table S4: reasons behind accepting taking COVID-19 vaccines available in Sudan. Table S5: reasons behind not taking COVID-19 vaccines as reported by participants. [file 3392667.f1.docx]

**Table S1. Knowledge of participants about COVID-19 vaccination**

| **Knowledge of participants** | **n** | **%** |
| --- | --- | --- |
| **Heard about COVID-19 vaccine** |  |  |
| Yes | 362 | 91.4 |
| No | 34 | 8.6 |
| Total | 396 | 100 |
| **COVID-19 vaccine safety** |  |  |
| Not completely safe given the limited time to test it | 211 | 54.2 |
| Safe because it is not the first SARS virus | 178 | 45.8 |
| Total | 389 | 100 |
| **Number of vaccine shots** |  |  |
| One shot | 43 | 10.8 |
| Two shots | 321 | 80.3 |
| More than two shots | 28 | 7 |
| Don't know | 8 | 2 |
| Total | 400 | 100 |

**Table S2. Perception of participants about COVID-19 vaccination**

| **Perception** | **n** | **%** |
| --- | --- | --- |
| **Is vaccination the key to stop the pandemic** | |  |
| Yes | 286 | 72.2 |
| No | 110 | 27.8 |
| Total | 396 | 100 |
| **Vaccination for recovered people from COVID-19 infection** | | |
| Yes | 244 | 61 |
| No | 88 | 22 |
| Don't know | 68 | 17 |
| Total | 400 | 100 |
| **Vaccination for COVID-19 infected people** | |  |
| Yes | 168 | 42 |
| No | 159 | 39.8 |
| Don't know | 73 | 18.3 |
| Total | 400 | 100 |

**Table S3. Acceptability of participants for COVID-19 vaccination**

| **Acceptability of the participants** | **n** | **%** |
| --- | --- | --- |
| **Accepting to get vaccinated** | | |
| Yes | 254 | 64 |
| No | 143 | 36 |
| Total | 397 | 100 |
| **Encourage family to take COVID-19 vaccine** | | |
| Yes | 265 | 67.4 |
| No | 125 | 31.8 |
| Don't know | 3 | 0.8 |
| Total | 393 | 100 |
| **When are you going to get vaccinated** | | |
| Already did | 90 | 22.9 |
| As soon as possible | 137 | 34.9 |
| After few months | 61 | 15.5 |
| Never | 105 | 26.7 |
| Total | 393 | 100 |

**Table S4. Reasons behind accepting taking COVID-19 vaccines available in Sudan**

| **Reasons behind accepting COVID vaccination** | **n** | **%** |
| --- | --- | --- |
| Worried about getting infected with COVID | 156 | 39 |
| Worried about my family getting infected | 144 | 36 |
| Social responsibility to get vaccinated | 71 | 17.8 |
| Worried about developing COVID complications | 63 | 15.8 |
| Not intending to take COVID vaccination | 16 | 4 |

**Table S5. Reasons behind not taking COVID-19 vaccines as reported by participants**

| **Reasons for not taking COVID vaccine** | **n** | **%** |
| --- | --- | --- |
| Inadequate data about safety | 84 | 29.4 |
| Concerned about the vaccination side effects | 66 | 23.1 |
| Against vaccination in general | 29 | 10.1 |
| Concerned about the vaccination efficacy | 25 | 8.7 |
| I avoid medications whenever possible | 21 | 7.3 |
| Not at high risk to develop complications from COVID infection | 19 | 6.6 |
| Concerned as low risk population, does not require COVID vaccine | 17 | 5.9 |
| Infected with COVID before | 12 | 4.2 |
| Vaccination is painful | 7 | 2.4 |
| Vaccination is inconvenient | 6 | 2.1 |
